# Instantaneous Degelling Thermoresponsive Hydrogel

**DOI:** 10.3390/gels7040169

**Published:** 2021-10-14

**Authors:** Noam Y. Steinman, Abraham J. Domb

**Affiliations:** The Alex Grass Center for Drug Design and Synthesis and Center for Cannabis Research, Faculty of Medicine, Institute of Drug Research, School of Pharmacy, The Hebrew University of Jerusalem, Jerusalem 91120, Israel; noam.steinman@mail.huji.ac.il

**Keywords:** PEG-PLA, thermoresponsive hydrogel, redox-sensitive

## Abstract

Responsive polymeric hydrogels have found wide application in the clinic as injectable, biocompatible, and biodegradable materials capable of controlled release of therapeutics. In this article, we introduce a thermoresponsive polymer hydrogel bearing covalent disulfide bonds. The cold aqueous polymer solution forms a hydrogel upon heating to physiological temperatures and undergoes slow degradation by hydrolytic cleavage of ester bonds. The disulfide functionality allows for immediate reductive cleavage of the redox-sensitive bond embedded within the polymer structure, affording the option of instantaneous hydrogel collapse. Poly(ethylene glycol)-b-poly(lactic acid)-S-S-poly(lactic acid)-b-poly(ethylene glycol) (PEG-PLA-SS-PLA-PEG) copolymer was synthesized by grafting PEG to PLA-SS-PLA via urethane linkages. The aqueous solution of the resultant copolymer was a free-flowing solution at ambient temperatures and formed a hydrogel above 32 °C. The immediate collapsibility of the hydrogel was displayed via reaction with NaBH_4_ as a relatively strong reducing agent, yet stability was displayed even in glutathione solution, in which the polymer degraded slowly by hydrolytic degradation. The polymeric hydrogel is capable of either long-term or immediate degradation and thus represents an attractive candidate as a biocompatible material for the controlled release of drugs.

## 1. Introduction

Biodegradable polymers are ubiquitous across the pharmaceutical industry. These materials often constitute the primary platform for the delivery of therapeutic agents, as their slow degradation in vivo allows for sustained drug release over an extended period of time without the need for subsequent removal of the delivery vehicle. ‘Smart’ polymers capable of responding to external stimuli were developed to afford targeted drug release based on the presence of the relevant stimulus. These materials bear functional groups capable of a quick response to small changes in temperature, pH, or light, which lead to a physical change in the polymer that triggers drug release [1].

Polymeric hydrogels describe polymers capable of absorbing large amounts of water to form a gel due to crosslinking of the polymer chains. Smart thermoresponsive hydrogels were developed to undergo a sol–gel transition in response to temperature variation [2]. This feature enables a liquid solution to be injected into a physiological environment and form a gel in situ at the point of injection due to the temperature change. The molecular structures of such polymers determine their gelling temperatures as well as important features such as biocompatibility [3,4].

Copolymers of poly (lactic-co-glycolic acid) (PLGA) and poly (ethylene glycol) (PEG) were described at length as biocompatible and biodegradable polymers capable of forming thermoresponsive hydrogels in water [5]. Triblock copolymers, either with the morphology PLA-PEG-PLGA or PEG-PLGA-PEG, are soluble in water and reversibly form hydrogels upon heating [6,7,8]. The gelling behavior of the hydrogels formed from these copolymers is affected by overall polymer molecular weight, the ratio between polymer blocks, and the concentration of the polymer in solution [3,9,10,11]. Hence, the rational design of each element of the polymer structure is critical to achieving desired gel properties.

Inspired by the crucial role of cysteine–cysteine bonds and cleavage thereof in biological processes, the disulfide (S–S) bonds were exploited in a variety of functional materials due to their redox responsiveness. Materials containing disulfide bonds may undergo specific cleavage under reductive conditions, particularly upon exposure to the reductive intracellular space, rendering redox-responsive functionality to polymers. Disulfide bonds were incorporated in polyurethanes to afford self-healing properties [12], in electrochemical polymers and devices [13,14], and in drug carriers to render reduction-specific crosslinking or drug binding [15,16,17] for the controlled release of antitumor drugs due to high concentrations of reducing agents in the tumor microenvironment [18], to reduce toxicity in gene delivery platforms [19,20,21,22], and in supramolecular polymer applications [23]. The opportunity to incorporate disulfide bonds in biocompatible thermoreversible hydrogels may render these materials dual-responsiveness to both temperature and reduction.

**Figure 1:**
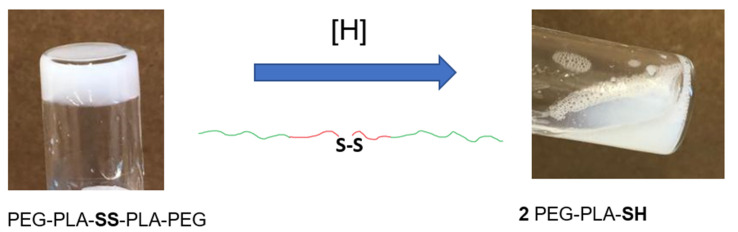
PEG-PLA-PEG triblock copolymers with embedded disulfide bonds immediately collapse upon addition of reducing agent NaBH_4_. Disulfide-bearing polymer hydrogels afford controlled hydrogel collapsibility due to S–S bond cleavage.

In this work, we describe a triblock PEG-PLA-PEG copolymer bearing one disulfide bond per molecule. The thermoresponsiveness of the polymer solution afforded a fully water-soluble material at cool temperatures, which formed a gel between 32 and 40 °C. The polymer possesses a cleavable disulfide bond capable of cleavage upon exposure to strong reducing agents, thereby rendering immediate hydrogel collapse. This proof of concept was displayed by the cleavage of the disulfide bond in the presence of hydride. A synthetic analog without a disulfide bond was stable as a hydrogel even under harsh reductive conditions. The work here represents, to the best of our knowledge, the first report of a thermoresponsive hydrogel capable of controlled instantaneous collapse, in this example upon exposure to reducing conditions (Figure 1).

## 2. Results

### 2.1. Rational Design

**Figure 2:**
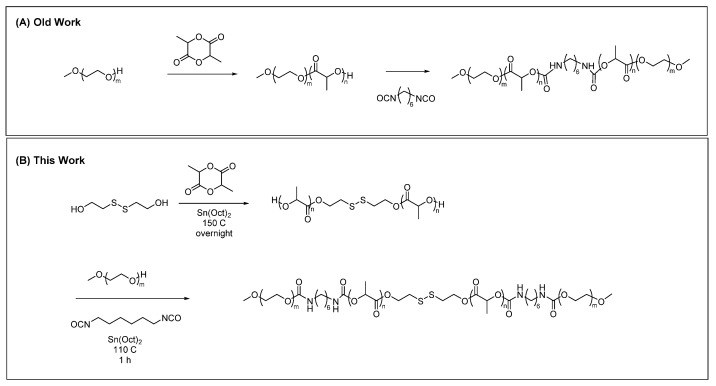
(**A**) Synthesis by Jeong et al. of a PEG-PLA-PEG triblock copolymer with urethane linkages [24]. (**B**) Synthesis of PEG-PLA-PEG with urethane linkages between polymer blocks and one embedded disulfide bond.

Jeong et al. first reported the synthesis of triblock copolymers containing a central poly (lactic acid) (PLA) block bearing two sidechains of poly (ethylene glycol) (PEG) [24]. The synthetic method reported there comprised of two steps. First, poly(ethylene glycol) methyl ether Mn = 550 (mPEG 550) was used as a macroinitiator of lactide polymerization (via ring-opening polymerization in the presence of stannous octoate catalyst) to form a diblock copolymer. Two hydroxyl termini of two distinct PLA blocks were subsequently linked by reaction with diisocyanate, forming urethane linkages (Figure 2A). Whereas the original research focused on polymers with sol–gel properties, which required cooling to move from sol form to gel morphology, subsequent reports focused on the development of sol–gel transitions, which occurred upon heating of the polymer solution. The enhancement of sol-to-gel properties from relying on cooling (gel at low temperatures and sol at high temperatures) to being responsive to heating (sol at room temperature and gel at elevated temperature) was crucial, as the practical application of such gels relies on the incorporation of active agents in a room-temperature solution, which upon injection and exposure to physiological temperatures forms a semi-solid gel capable of extended release of the active agent. These sol–gel thermosensitive hydrogels were based on an amorphous PLA polymer block (as opposed to crystalline poly (L-lactic acid) polymer blocks employed in the original work). Gel properties, biodegradability, and drug release were all subsequently optimized, leading to over two decades of research and application of injectable sol–gel biodegradable polymer solutions [6,11,25].

The PEG-PLA-PEG triblock copolymer described here is an analog of the original work performed by Jeong and co-workers. Its chemical structure bears the modular hydrophobic and hydrophilic units at the appropriate ratios for effecting sol–gel transitions near physiological temperatures, with the added benefit of one embedded disulfide bond. In doing so, the ‘smart’ hydrogel was imbued with secondary responsiveness to reduction agents in addition to its thermal responsivity. The hydrogel resultant thereof was therefore capable of injection as a liquid at ambient temperatures, gelling upon moderate heating, and subsequent collapse of the 3D gel structure upon exposure to strong reducing agents [26]. The following is a description of the synthesis and characterization of the novel triblock copolymer with a comprehensive study of the gel properties of aqueous solutions prepared thereof.

### 2.2. Synthesis

In a similar fashion as the original work on PEG-PLA-PEG triblock copolymers, several synthetic steps were employed in order to incorporate a disulfide bond into the triblock copolymer structure. In the first synthetic step, 2-hydroxyethyl disulfide was used as the initiator of DL-lactide polymerization. In doing so, an amorphous DL-poly(lactic acid) (DL-PLA) was obtained bearing one embedded disulfide bond (PLA(SS)). In order to complete the triblock copolymer synthesis, mPEG with hydroxyl termini were linked to either terminus of the amorphous PLA diol by a 2:2:1 molar reaction between mPEG diisocyanate and PLA(SS), affording urethane bridges between PEG and PLA polymer blocks with an overall PEG-PLA-SS-PLA-PEG copolymer structure (Figure 2B).

### 2.3. Characterization

The formation of PLA(SS) and PEG-PLA-SS-PLA-PEG polymers was confirmed spectroscopically by 1H NMR. Polymer molecular weights (MW) were estimated by size-exclusion chromatography (SEC). The presence of a disulfide bond was confirmed by elemental analysis (CHNS).

#### 2.3.1. ^1^H NMR

**Figure 3:**
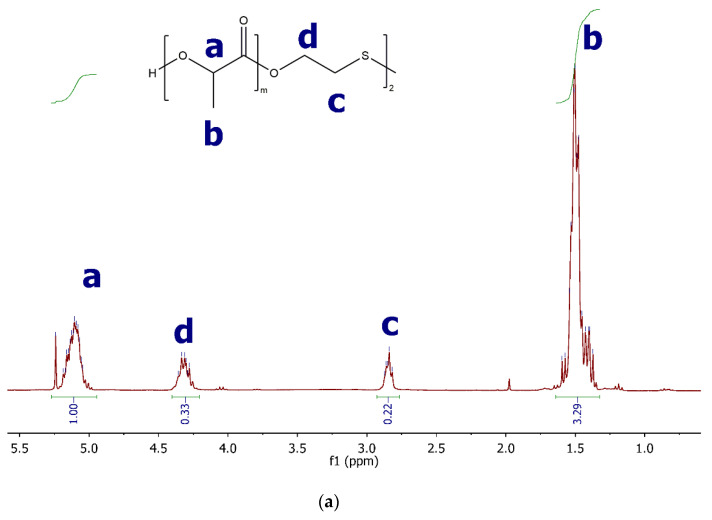

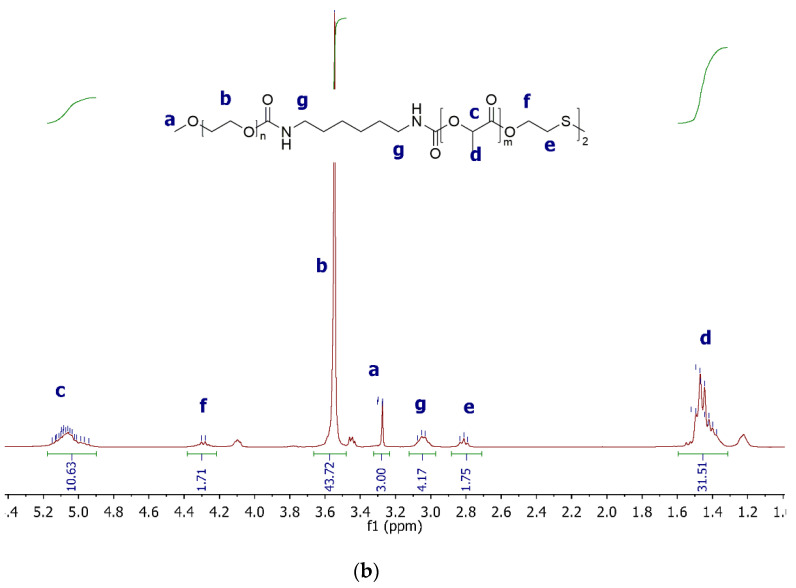
(**a**) 1H NMR spectra of PLA(SS) with peak assignments.; (**b**) 1H NMR spectra of PEG-PLA-SS-PLA-PEG with peak assignments.

The structures of both PLA(SS) and PEG-PLA-SS-PLA-PEG were confirmed by 1H NMR (Figure 3) by observing all available proton peaks. For PLA(SS), in addition to characteristic multiplets for lactic acid at 5.2 and 1.5 ppm [3], a downfield shift of 2-hydroxyethyl disulfide CH_2_ protons was observed upon polymerization in the PLA(SS) spectrum, indicating the formation of the ester bond at the termini of the disulfide reagent. No other peaks were observed in the spectrum, indicating a pure substance.

Upon linkage to PEG, no chemical shifts were observed for peaks from PLA(SS), confirming the stability of the PLA block of the copolymer to the reaction conditions. Urethane linkages were confirmed by a peak at δ 3.05 with 2x integration relative to the CH_2_ peak at the disulfide, as expected. A characteristic PEG singlet was observed at δ 3.55, and all integrations were calculated relative to CH_3_ of mPEG. No other peaks were observed in the spectrum, indicating a pure substance (Figure 3).

#### 2.3.2. Molecular Weight Determination

Weight average (Mw) and number average (Mn) molecular weights of PLA(SS) and PEG-PLA-SS-PLA-PEG were estimated by SEC by comparing to polystyrene standards. Polydispersity (PDI) values were calculated from these measured values. Mn was further calculated by ^1^H NMR peak integrations using known integration values (CH_2_ of 2-hydroxyethyl disulfide for PLA(SS), CH_3_ of mPEG for triblock PEG-PLA-PEG copolymer). Mn values calculated by NMR closely reflected the expected chain lengths based on feed ratios of starting materials, indicating full conversion from PLA(SS) to triblock copolymer with PEG. Estimated values provided by SEC indicated increased polymer MW upon copolymerization with PEG by urethane bridge, and only a slight increase in PDI values indicated polymers with high purity (Table 1).

### 2.4. Rheometry Studies

**Figure 4:**
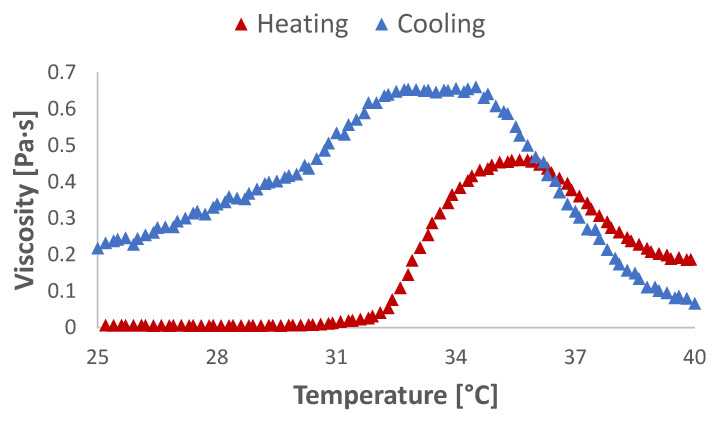
Heating (red) and cooling (blue) viscosity curves of 25% w/w PEG-PLA-SS-PLA-PEG aqueous solutions.

The PEG-PLA-SS-PLA-PEG triblock copolymer formed a free-flowing solution in double-distilled water at room temperature (25% w/w). Upon incubation at physiological temperature, the solution formed a hydrogel which did not flow upon inversion of the test tube [3,4]. In order to evaluate the flow behavior of the copolymer solution and determine the temperature at which the sol–gel transition occurred, the viscosity of copolymer solutions was measured as a function of temperature (Figure 4). Upon heating, an increase in viscosity was observed beginning at ~32 °C with a maximum 70-fold increase in viscosity at 36 °C. The high viscosity values were stable through physiologically relevant temperatures. Upon cooling of the hydrogel, the opposite trend was observed, indicating reversibility of the thermoresponsive system. This flow behavior indicates a polymeric solution capable of injectability at ambient temperatures (<30 °C) with temperature-induced gelling occurring upon exposure to physiological temperatures.

### 2.5. Degradation Studies

Degradation of injectable implants such as triblock copolymer hydrogels is an important parameter for the determination of the hydrogel’s potential applicability to the clinic [27]. The stability of the hydrogel under physiological conditions affects the therapeutic window and controlled release profile of incorporated drugs, and biodegradation of the hydrogel matrix excludes the necessity for post-injection removal of the material, allowing it to slowly degrade and be eliminated by the body.

In the case of the PEG-PLA-PEG copolymer with an embedded disulfide bond, two modes of degradation are possible. One degradable bond is the repeating ester bond throughout the PLA polymer block, known to be susceptible to hydrolytic cleavage [28]. Secondly, the disulfide bond may degrade upon exposure to reducing agents. Hydrolytic degradation of the ester bonds was expected to occur slowly over time, allowing for slow degradation of the hydrogel matrix. The disulfide bond, however, was expected to remain intact under in vitro physiological conditions, degrading only upon exposure to reducing agent. Due to its position in the center of the polymer chain, cleavage of the disulfide bond should lead to an immediate release of water retained in the hydrogel matrix due to rupture of the triblock copolymer structure. In order to investigate these effects, degradation of PEG-PLA-SS-PLA-PEG triblock copolymer was studied in water or in the presence of reducing agents such as sodium borohydride (NaBH_4_) or L-glutathione.

**Figure 5:**
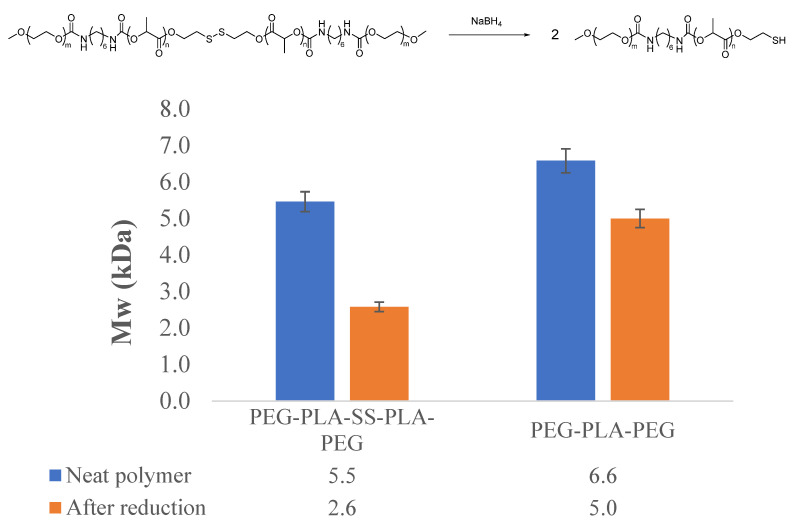
Sensitivity of the embedded disulfide bond in PEG-PLA-PEG triblock copolymer was confirmed by reaction with NaBH_4_ and subsequent reduction in molecular weight by half. A chemical analog lacking the disulfide bond retained most of its molecular weight under the same conditions, indicating the role of disulfide bond cleavage in molecular weight reduction. Error bars represent the standard deviation of experiments performed in triplicate.

In order to confirm the redox reactivity of the disulfide bond embedded in the PEG-PLA-PEG triblock copolymer structure, NaBH_4_, a strong hydride-based reducing agent, was added to the hydrogel matrix. The hydride was expected to reduce the disulfide bond, affording diblock PEG-PLA copolymers with thiol end groups (Figure 5). Indeed, a reduction in polymer molecular weight to half was observed upon reaction with hydride, indicating the cleavage of the reduction-sensitive disulfide bond in the triblock copolymer structure (Figure 5). A chemical analog of the triblock copolymer was prepared using 1,6-hexanediol as the initiator of PLA synthesis, resulting in PEG-PLA-PEG triblock copolymer without a disulfide bond (experimental details and spectral data available as Supplementary Materials, Figures S1 and S2). The molecular weight of this polymer was only slightly reduced upon exposure to the same hydride reagent, thereby confirming the role of the disulfide bond in a molecular weight reduction in the original polymer (Figure 5).

**Figure 6:**
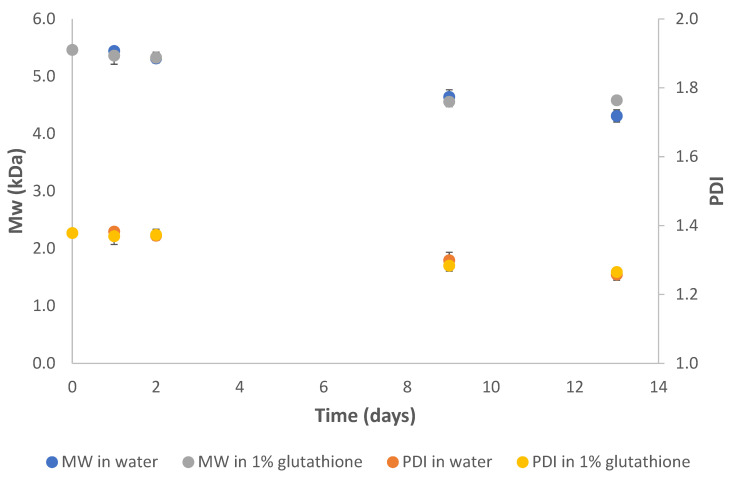
Degradation of PEG-PLA-SS-PLA-PEG hydrogel in distilled water or 1% glutathione solution displayed a slow hydrolytic degradation for two weeks with near-constant PDI values.

In order to evaluate the in vitro degradation of PEG-PLA-SS-PLA-PEG hydrogels, polymer molecular weight was monitored for two weeks at physiological temperature (Figure 6). The hydrogel remained stable for this period, after which the molecular weight was sufficiently reduced to inhibit the water retention capabilities of the hydrogel, resulting in the release of water from the 3D hydrogel structure. A hydrogel containing 1% L-glutathione displayed no effect on the degradation rate of the polymer. We hypothesize that the rigidity of the hydrogel prevented the thiol reducing agent from reducing the disulfide bond embedded within the triblock copolymer structure. The near-constant PDI and slow Mw reduction in the polymer under either condition indicates a hydrolytically degradable polymeric hydrogel with stability in the presence of thiol reducing agents (Figure 6).

## 3. Discussion

**Figure 7:**
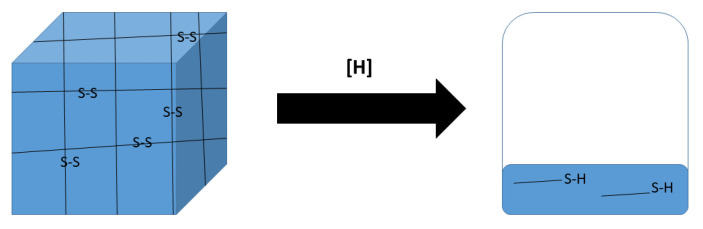
Three-dimensional structure of a physically crosslinked hydrogel may be instantaneously collapsed by exposure to reducing conditions.

Polymer systems that undergo phase transitions in response to environmental stimuli such as temperature and pH have been widely investigated for drug delivery and tissue engineering applications [29]. The aqueous copolymer solution presented in this work represents a programmable biodegradable responsive polymer gel in which its gelling properties can be additionally controlled by a cleavable S–S bond triggered to cleave and change the gel properties. The polymer is a non-crosslinked triblock copolymer of polyethylene glycol (PEG) blocks and a hydroxy acid aliphatic polyester block that possesses thermo-responsive properties where the polymer is fully water soluble at temperatures between 0 °C and about 30 °C and gels at body temperature (37 °C). In addition to hydrolytically degradable ester bonds, the polymer possesses, as mentioned above, a cleavable bond that breaks upon application of an external reductive trigger that is faster than the hydrolysis of the ester bonds of the polyester block. The cleavage of this bond results in the collapse of the gel to dissolution in water with no remaining gel properties (Figure 7). This class of polymer may have medical use as a device for drug carrying or cell support and delivery. For drug or cell delivery, the rate of de-gelation can be programmed so that after its formation at body temperature, the gel may gradually or instantly lose/loosen its gel properties to allow faster release from the gel of the entrapped cells or drugs. The change in gel properties, after initial gelation at the site of action, can be tailored according to the need for a specific application, either immediately or gradually, based on the presence of reducing agents. Of specific interest is the delivery of therapeutic stem cells carrying anticancer cargo at cancerous tissue. The cells may be delivered to the site of treatment as a solution that instantly gels on the target tissue due to temperature change and starts to lose its gel properties to allow the cells to move towards cancerous tissue due to the increased neoplastic concentration of reducing agents [18].

Several important parameters are involved in the development of these systems, including robust synthetic methods, defined stimuli-responsive gelation, and degradation for application in vivo. The physical crosslinking mechanism of PEG-PLA-PEG copolymers hydrogel formation permits the additional advantage of reversibility, as no chemical bonds must be cleaved in order to re-obtain the polymer in solution [30]. However, to date, there have been no reports of non-crosslinked thermoresponsive polymer gels that possess all of the following qualities: (1) are water-soluble below physiological temperatures, (2) form a semi-solid continuous gel at physiological temperatures, and (3) possess breakable bonds that permit an immediate programmable change in gel properties following exposure to an external trigger. The PEG-PLA-PEG triblock copolymers with embedded disulfide bond described here represent such material, with gelling properties suitable for physiological application and susceptibility to water leakage upon exposure to reducing agents. The proof of concept afforded by cleavage with a hydride reagent represents an interesting option for the development of reduction-sensitive hydrogels capable of instantaneous collapse upon exposure to reducing agents.

## 4. Conclusions

Thermoresponsive hydrogels capable of immediate collapse upon exposure to strong reducing agents represent an interesting injectable platform for the controlled release of drugs. At ambient temperatures, the polymer dissolved in water (25%) exists as a free-flowing solution that forms a hydrogel upon heating. The disulfide bond embedded in the polymer structure induces a controlled hydrogel collapse by the addition of NaBH_4_. The hydrogel is otherwise stable for a period of over two weeks. Hydrolytic degradation of ester bonds results over time in hydrogel collapse into safe by-products. The hydrogels described here have the potential to be applied as delivery vehicles for the slow release of therapeutics with immediate release induced upon contact with reducing agents.

## 5. Materials and Methods

### 5.1. Materials

Stannous octoate, hexamethylene diisocyanate, and poly(ethylene glycol) monomethyl ether were purchased from Sigma Aldrich (Rehovot, Israel). Lactide was purchased from Purac Biochem BV (Gorinchem, The Netherlands). Solvents were purchased from Bio-Lab Ltd. (Jerusalem, Israel).

### 5.2. General Methods

Chemical reactions were performed in dry glassware under N_2_ gas. ^1^H NMR spectra were obtained on a Varian 300 MHz spectrometer with CDCl_3_ as the solvent and tetramethylsilane as the shift reference. The molecular weights of the polymers were estimated using a gel permeation chromatography (GPC) system consisting of a Waters 1515 Isocratic HPLC pump with a Waters 2410 Refractive Index Detector and a Rheodyne (Cotati, CA, USA) injection valve with a 20 mL loop (Waters, Milford, MA, USA). Samples were eluted with CHCl3 through a linear Styragel HR4E column (7.8 × 300 mm i.d.; Waters) at a flow rate of 1 mL/min. The molecular weights were determined relative to polystyrene standards (Polyscience, Warrington, PA, USA) using a Breeze computer program.

### 5.3. Synthesis

PEG-PLA-PEG triblock copolymer with disulfide bond was prepared in two steps. First, 50 µL of a 10% solution of stannous octoate in dichloromethane (DCM) was added to a melt of 2-hydroxyethyl disulfide (0.13 g, 0.82 mmol) and D,L-lactide (2.4 g, 16 mmol). The solvent was allowed to evaporate, and the vial was purged with N_2_. The mixture was stirred at 120 °C for 2 h, followed by overnight stirring at 150 °C. The crude polymer was taken up in DCM and precipitated into ether to afford PLA disulfide PLA(SS) as a pure substance. ^1^H NMR (300 MHz, CDCl_3_, δ): 5.24–5.05 (m, LA), 4.36–4.28 (m, CH_2_ α-ester), 2.87–2.82 (m, CH_2_ α-disulfide), 1.59–1.37 (m, LA). Anal. Calcd: C, 46.74; H, 5.91; S, 3.80. Found: C, 48.00; H, 5.70; S, 4.42.

In the next step, hexamethylene diisocyanate (0.11 mL, 0.68 mmol) was added to a melt of PLA(SS) (1.0 g, 0.70 mol) and poly(ethylene glycol) methyl ether Mn = 550 (0.34 mL, 0.68 mmol). One drop of stannous octoate was added, and the mixture was stirred at 110 °C for one hour. The crude product was dissolved in cold water, filtered, and lyophilized to afford PEG-PLA(SS)-PEG as a pure substance. Proton nuclear magnetic resonance (^1^H NMR) (300 MHz, CDCl_3_, δ): 5.15–4.94 (m, LA), 4.30–4.28 (m, CH_2_ α-ester), 4.11–4.08 (m, PEG α-urethane), 3.55 (s, PEG), 3.28 (s, CH_3_), 3.07–3.03 (m, CH_2_ α-urethane), 2.83–2.79 (t, *J* = 6 Hz, CH_2_ α-disulfide), 1.50–1.38 (m, LA). Anal. Calcd: C, 50.53; H, 7.12; N, 2.05; S, 1.88. Found: C, 51.09; H, 7.22; N, 1.99; S, 2.27.

### 5.4. Rheological Studies

Sol–gel transitions were measured on 25% w/w polymer solutions in double-distilled water (DDW) by rotational tests on a Physica MCR 101 rheometer (Anton Paar, Austria) as a function of temperature from 25 to 45 °C.

### 5.5. Degradation Studies

25% w/w polymer solutions were prepared in DDW or in 1% solutions of either reduced L-glutathione or NaBH_4_. Solutions were incubated at 37 °C, and molecular weight was monitored by GPC for two weeks.

## Figures and Tables

**Table 1:** Molecular weight determination of disulfide PLA and disulfide-containing PEG-PLA-PEG copolymer.

Polymer	PLA(SS)	PEG-PLA-SS-PLA-PEG
Expected MW ^1,2^	1.53	1.96
Mn (^1^H NMR) ^1,3^	1.46	3.11
Mn (SEC) ^1,4^	2.65	4.24
Mw (SEC) ^1,4^	2.85	5.97
PDI ^5^	1.07	1.41

^1^ Values given in kDa. ^2^ Based on feed ratios of starting materials. ^3^ Calculated based on peak integrations of known value. ^4^ Calculated from size-exclusion chromatography (SEC). ^5^ Calculated from SEC as Mw/Mn.

## Data Availability

Not applicable.

## Supplementary Materials

The following are available online at https://www.mdpi.com/article/10.3390/gels7040169/s1, Experimental details: Figure S1: ^1^H NMR spectrum of PLA analog; Figure S2: ^1^H NMR spectrum of PEG-PLA-PEG analog.
