# Supplementary material for: Instantaneous Degelling Thermoresponsive Hydrogel
Source: Gels. 2021 Oct 14;7(4):169. doi: 10.3390/gels7040169 (PMC8544516; doi:10.3390/gels7040169)
Supplement: Supplementary file 1 [file gels-07-00169-s001.zip › gels-1429322-supplementary.pdf]

Instantaneous degelling thermoresponsive hydrogel

Noam Y. Steinman [1] and Abraham J. Domb [2,*]

[1] The Alex Grass Center for Drug Design and Synthesis and Center for Cannabis Research and the Institute of Drug Research, School of Pharmacy-Faculty of Medicine, The Hebrew University of Jerusalem, Jerusalem, 91120, Israel; noam.steinman@mail.huji.ac.il

[2] The Alex Grass Center for Drug Design and Synthesis and Center for Cannabis Research and the Institute of Drug Research, School of Pharmacy-Faculty of Medicine, The Hebrew University of Jerusalem, Jerusalem, 91120, Israel; avid@ekmd.huji.ac.il

* Correspondence: avid@ekmd.huji.ac.il

Supporting Information

Synthesis of PEG-PLA-PEG analogue without disulfide bond

PEG-PLA-PEG analogue without disulfide bond was prepared in two steps. First, 50 μL of a 10% solution of stannous octoate in dichloromethane (DCM) was added to a melt of 1,6-hexandiol (0.26 g, 2.2 mmol) and D,L-lactide (3.1 g, 21 mmol). Solvent was allowed to evaporate and the vial was purged with N2. The mixture was stirred at 120°C for 2 h, followed by overnight stirring at 150 °C to afford PLA as a pure substance. Proton nuclear magnetic resonance (1H NMR) (300 MHz, CDCl3, δ): 5.15-5.10 (m, LA), 4.13-4.07 (m, CH2 α-ester), 1.62-1.40 (m, LA).

In the next step, hexamethylene diisocyanate (0.59 mL, 3.7 mmol) was added to a melt of PLA (2.7 g, 1.8 mmol) and poly(ethylene glycol) methyl ether Mn = 550 (1.9 mL, 3.1 mmol). One drop of stannous octoate was added, and the mixture was stirred at 110 °C for one hour to afford PEG-PLA-PEG as a pure substance. Proton nuclear magnetic resonance (1H NMR) (300 MHz, CDCl3, δ): 5.15-5.10 (m, LA), 4.18-4.08 (m, CH2 α-ester, α-urethane), 3.61 (s, PEG), 3.34 (s, CH3), 3.16-3.07 (m, CH2 α-urethane), 1.56-1.46 (m, LA).

^{1}H NMR of PEG-PLA-PEG analogue without disulfide bond

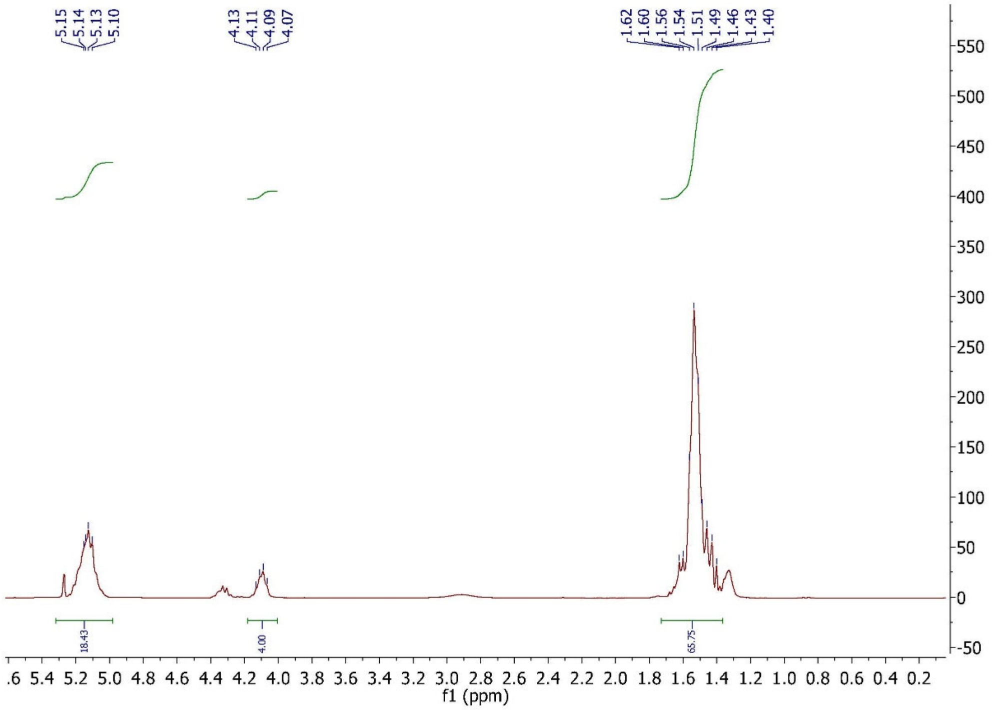

Figure S1. ^{1}H NMR spectrum of PLA analogue.

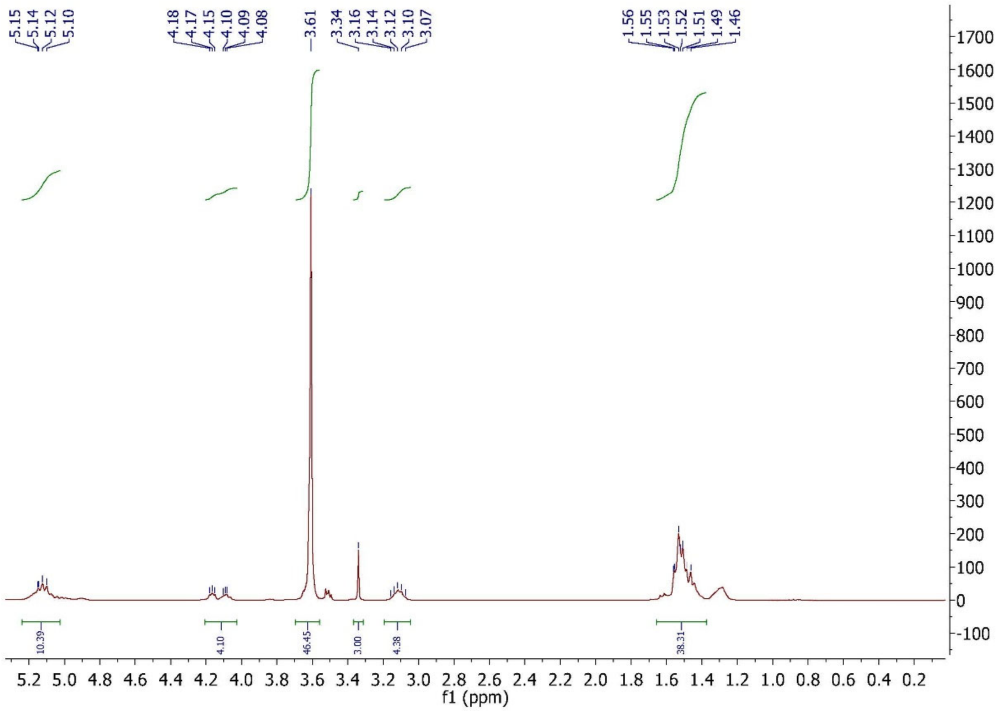

Figure S2. ^{1}H NMR spectrum of PEG-PLA-PEG analogue.
